# Suicide attempt by a cancer caregiver: A husband who attempted suicide two months after his wife was diagnosed with advanced stomach cancer

**DOI:** 10.1017/S1478951526101667

**Published:** 2026-02-02

**Authors:** Mayumi Ishida, Yoshitaka Ooya, Chiaki Kawanishi, Tomonori Kashiwagi, Hideki Onishi

**Affiliations:** 1 Department of Psycho-oncology, Saitama Medical University International Medical Center, Saitama, Japan; 2 Departments of Emergency and Acute Medicine, Saitama Medical University International Medical Center, Saitama, Japan; 3 Department of Neuropsychiatry, Sapporo Medical University Graduate School of Medicine, Hokkaido, Japan

**Keywords:** Suicide attempt, cancer, caregiver, family, caregiver’s clinic

## Abstract

**Objectives:**

The families of cancer patients experience many forms of distress, as a result of their loved one’s cancer diagnosis. However, there have been no reports of suicide attempts of caregivers directly linked to the diagnosis of advanced cancer in a family member.

**Methods:**

We reported a caregiver who attempt suicide two months after his wife was diagnosed with advanced cancer.

**Results:**

The subject was a 69-year-old male who had been caring for his wife, diagnosed with advanced stomach cancer, for two months. The patient’s husband, acting as her caregiver, was referred by his wife (a cancer patient) to meet with a nurse. He reported insomnia and a desire for hastened death. Despite repeated recommendations for specialized care at a caregiver clinic, he declined. Following an argument with his wife at home, he felt unable to cope and attempted suicide. The husband had no psychiatric history but had a history of colon cancer. After the attempt suicide, he began visiting the “Caregivers’ Clinic,” where he received ongoing psychological support that continued until the death of his wife.

**Significance of results:**

In cancer care, it is essential to continuously assess not only the patient’s suicide risk, but also that of closely related family members.

## Introduction

It is well-known that the families of cancer patients experience physical, psychological, social, and existential burdens as a result of their loved ones being diagnosed with cancer (Braun et al. 2007; Murray et al. 2010; Sjovall et al. 2010; McAndrew et al. 2023). Therefore, family members are said to be “second order patients” and require treatment and care (Lederberg 1998). It has been shown that caregivers as relatives of patients with advanced cancer experience a high caregiver burden (Van Roij et al. 2021).

Thus, our hospital has set up a Caregiver’s Clinic to provide care for the families of cancer patients (Ishida and Onishi 2021).

In recent years, psychological distress and suicide-related behaviors have been reported in cancer patients (Fang et al. 2012; Yamauchi et al. 2014), highlighting the importance of care that includes suicide prevention. However, as mentioned above, while there are reports of depression among family members, there are very few reports of suicide behavior. Therefore, the accumulation of case studies is necessary.

Herein, we report a case of attempted suicide by the spouse of a cancer patient during the course of caregiving, focusing on the psychological changes experienced by the family, as well as the responses leading up to the event.

## Case report

### The cancer patient’s time course

A 67-year-old woman was diagnosed with gastric cancer. She was scheduled for a total gastrectomy, but during laparotomy, invasion into the colon was observed, and the surgery was aborted. On the same day, the attending physician informed the patient and her family that the cancer was Stage IV and that her prognosis without treatment was approximately 1 month. The physician also recommended chemotherapy be started as soon as possible, and S-1 oral treatment (Sakata et al. 1998) was initiated.

Seven weeks after the diagnosis, although the tumor marker levels had decreased, the patient’s anxiety about the future intensified. Consequently, the attending physician referred her to the psycho-oncology department.

During the consultation, she explained the events that had occurred and her emotional distress. She was diagnosed with adaptation response disorder and continued to receive care thereafter. At the consultation the following week (week 8), she expressed concern for her husband, saying, “My husband is more depressed than I am.”

## The spouse’s (husband’s) time course

The spouse was a 69-year-old male. Five weeks after the diagnosis, he had a consultation with a nurse at his wife’s suggestion. During the consultation, he said, “I can’t sleep” and “I might as well be dead.” Although the nurse recommended and encouraged the patient’s husband to visit the “Caregiver’s Clinic” several times, he continued to decline. However, at week 11, he attempted to hang himself at home and was transported to our hospital’s emergency and acute medical care center.

After the suicide attempt, additional background information regarding the husband became clear. He had undergone treatment for colorectal cancer 18 years previously, with no recurrence. Furthermore, the cancer was discovered following a screening recommended by his wife. No history of psychiatric disorders, or alcohol or drug abuse was noted. Further, he did not show any problems with interpersonal relationships prior to the suicide attempt. The couple have been married for over 20 years, with their 2 children both living independently. Their relationship is good, and they have no financial problems.

From when she was diagnosed with advanced gastric cancer, he began to think, “They could detect and treat my colorectal cancer early because my wife pushed me to get screened, but I never encouraged my wife to get screened. It’s my fault that she got cancer.”

Later, when the scheduled surgery was not performed, he became even more depressed and began telling people around him, “If my wife dies, it’s all over for me. I’ll kill myself too.” On the day of his attempted suicide, he had an argument with his wife, who told him, “You are responsible for me having stomach cancer.” The husband thought, “If my wife says that, there’s no point in me living,” and “There’s nothing I can do about it now.” Twenty minutes later, he attached 10 kg dumbbells to both of his feet and attempted to hang himself with a rope in his garden. His wife, who heard a noise, soon found him and was able to save him. After several days of psychiatric evaluation following his hospitalization, he strongly denied having suicidal thoughts. To prevent recurrence, we held repeated in-depth interviews with him, his family, and the psychiatrists regarding numerous measures, including physical and psychological care as well as environmental adjustments. A follow-up psychiatric appointment was scheduled shortly thereafter, and he was subsequently discharged.

Subsequently, he was referred to the Caregiver’s Clinic, where he began receiving psychological support. At the Caregiver’s Clinic, information regarding the wife’s medical condition was regularly shared, and realistic caregiving expectations were discussed as needed. Efforts were made to avoid excessive physical and psychological burden on the husband, and communication was also maintained with the couple’s daughters and, at times, the patient herself. No pharmacological treatment was administered; instead, he received intensive support through psychotherapy. Two years after her diagnosis, his wife passed away at home in accordance with her wishes.

Since then, he has not developed any psychiatric disorders and there has been no recurrence of suicidal ideation. He continues to work to this day.

## Discussion

This case involves a husband who experienced multiple instances of bad news, including his wife’s diagnosis of advanced gastric cancer and her poor prognosis, which led to a decline in his mental health and eventually a suicide attempt following an argument. As in this case, feelings of despair, as well as depression and hopelessness, are known risk factors for suicidal behavior (Ribeiro et al. 2018; Turecki and Brent 2016), and suicide attempts have also been reported in patients with mood or adjustment disorders (Yamada et al. 2007).

In light of the husband’s psychological burden, his thinking, such as blaming himself for not encouraging his wife to undergo screening, can be seen as signs of extreme cognitions and psychological tunnel vision, suggesting he may have already been experiencing a mental disorder at the time. In this context, his wife’s comment, “You are responsible for this,” was interpreted through cognitive distortions, leading him to believe her words were entirely justified and this intense sense of guilt became a major trigger for his suicide attempt.

The husband had been encouraged to visit the Caregiver’s Clinic several weeks before his suicide attempt. However, many caregivers have difficulty in accessing mental health services even when they meet the criteria for a psychiatric diagnosis (Vanderwerker et al. 2005), and many people who die by suicide do not seek medical care (Owens et al. 2004).

Depression, hopelessness, and the desire for hastened death, which have all been documented among patients themselves (Breitbart 2000), can also occur in caregivers.

Palliative care should naturally also be provided to family members, and self-care for relatives is important for the wellbeing of the relatives themselves and may also be beneficial for the cancer patient (Van Roij et al. 2021). Thus, effective, concrete methods for doing so are now being developed (Greer et al. 2020; Applebaum et al. 2021; Applebaum et al., 2024). This support extends beyond abstract concepts or psychological issues such as anticipatory grief, and it is also important to identify forms of care that enable caregivers to assume meaningful roles tailored to the patient’s changing condition (McAndrew et al. 2024) and coordination, including information sharing with the primary physician, is also essential.

Excessive psychological consideration of the husband following his suicide attempt may result in missed opportunities to provide care as a caregiver, which could lead to prolonged regret and distress after bereavement (Ishida et al. 2012). Moreover, assertive care management is effective at reducing the incidence of repetition of suicide attempts (Kawanishi et al. 2014), ongoing assessment that considers the caregiver’s medical history and suicide risk, along with thorough documentation of the caregiver’s status, can enable continued support even after the patient’s death.

In cancer care, it is essential to continuously assess not only the patient’s suicide risk but also that of closely related family members.
